# Peripartum cardiomyopathy in a 28-year-old patient—case report

**DOI:** 10.3389/fcvm.2025.1666816

**Published:** 2025-09-03

**Authors:** Katarzyna Hajduk, Aleksandra Gil, Wojciech Papis, Gabriela Skórska, Joanna Śliwka, Szymon Pawlak, Tomasz Hrapkowicz

**Affiliations:** ^1^ Student Scientific Association of Pediatric Cardiac Surgery, Department of Cardiac, Vascular and Endovascular Surgery and Transplantology, Medical University of Silesia in Katowice, Katowice, Poland; ^2^ Department of Cardiac, Vascular and Endovascular Surgery and Transplantology, Medical University of Silesia in Katowice, Silesian Center for Heart Diseases, Zabrze, Poland

**Keywords:** peripartum cardiomyopathy (PPCM), pregnancy-related cardiomyopathy, heart tranplantation, heart faiIure, postpartum heart failure

## Abstract

Peripartum cardiomyopathy (PPCM) is a rare but serious form of heart failure that occurs toward the end of pregnancy or in the earlypostpartum period. We present the case of a 28-year-old woman who developed severe biventricular heart failure and cardiac arrest shortly after childbirth. Despite treatment with mechanical circulatory support with veno-arterial extracorporeal membrane oxygenation (VA-ECMO) and intra-aorticballoon pump (IABP), her condition did not improve, and she was urgently referred for heart transplantation. Histological examination of theexplanted heart revealed noncompaction cardiomyopathy (NCC), a rare structural heart disease that may mimic PPCM. This case highlights the challenges in diagnosing PPCM and the importance of early intervention, including advanced support and transplant, in cases with poor recovery prospects.

## Introduction

Peripartum cardiomyopathy (PPCM) is a form of idiopathic congestive heart failure involving a reduced left ventricular ejection fraction (LVEF) occurring toward the end of pregnancy or within several months postpartum, after other potential causes have been excluded (1). The worldwide incidence of PPCM is reported to range from 1/100 to 1/2,000 postpartum women (2). Several factors are believed to elevate the risk of PPCM, including maternal age above 30 years, women of African descent, preeclampsia, pregnancy-induced hypertension, and multifetal gestations (3). The prognosis of PPCM is determined by the severity of myocardial dysfunction and the timing of initiating appropriate treatment. In certain cases, orthotopic heart transplantation remains the only viable treatment option.

## Case description

A 28-year-old woman, following her first pregnancy and delivery, was urgently admitted to a cardiology clinic after experiencing sudden cardiac arrest secondary to acute heart failure. Upon admission, the patient reported progressive asthenia, right upper quadrant pain, nausea, and emesis. The patient was diagnosed with severe biventricular heart failure (EF = 15%), bilateral pleural effusions, free peritoneal fluid, hepatomegaly and liver dysfunction. Echocardiography revealed atrial flutter with a heart rate of 160 bpm.

Sudden cardiac arrest occurred due to a bradyasystolic mechanism. It was decided to implant VA-ECMO along with IABP. After the procedure patient was transferred to the intensive care unit (ICU). On admission, remained unconscious, without consensual pupillary reflexes, and scored 5 on the Glasgow Coma Scale. Laboratory findings revealed hypocapnia, hyperoxia, severe metabolic acidosis, and complex coagulation abnormalities involving multiple pathways. TEE revealed a non-contractile heart, a dilated left cardiac chamber containing an intracavitary thrombus, 7–10 mm of pericardial effusion, and bilateral pleural exudates. Following airway suctioning, the patient regained coherent logical contact. The patient was subsequently placed in an induced coma (MF + Mid). On the second day of hospitalization, while on ECMO support, the patient's condition remained critical, with cardiogenic shock and multiorgan failure involving the cardiovascular, respiratory, hepatic, and renal systems. TEE demonstrated global myocardial hypokinesis, severe mitral regurgitation, absence of aortic valve opening, a thrombus within the left ventricular cavity, and features consistent with hypertrophic cardiomyopathy. The presence of an intracardiac thrombus excluded the possibility of active LV unloading or initiating long-term mechanical circulatory support (LT-MCS). According to information provided by the family, the patient may have experienced myocarditis during adolescence.

Considering the patient's clinical condition, the probability of myocardial recovery—regardless of the underlying cause of cardiomyopathy—was deemed low, while the risk of stroke remained significantly high. Given the life-threatening circumstances, the patient was urgently qualified for heart transplantation as a life-saving measure.

On the fifth day of hospitalization, the patient underwent orthotopic heart transplantation. The procedure was performed under cardiopulmonary bypass, during which ECMO support was discontinued and the cannulas were surgically removed. After operation no signs of cellular rejection were observed. In the early post-operative period she required support with IABP, which was discontinued on the seventh day of hospitalization.

Histological analysis confirmed NCC, raising the possibility that the initial presentation may have been unmasked by peripartum hemodynamic stress rather than idiopathic PPCM.

18 months after heart transplantation, an endomyocardial biopsy was performed via the right internal jugular vein. Histopathological examination of the myocardial tissue samples revealed no signs of cellular rejection. The patient reports no deterioration in physical performance and remains in New York Heart Association (NYHA) functional class I. Echocardiographic assessment demonstrated normal function of the transplanted heart. Laboratory tests showed no new significant abnormalities. Mild leukopenia and anemia persist, along with an abnormal plasma lipid profile.

## Discussion

The association between the peripartum period and cardiomyopathy has been recognized for over 80 years. In 1937, Gouley et al. described seven pregnant women with acute heart failure. Autopsies revealed myocardial hypertrophy with areas of fibrosis and necrosis (3). Thirty-four years later, the concept of postpartum cardiomyopathy (PPCM) was introduced. Demakis and Rahimtoola proposed the first diagnostic criteria for this condition (3, 4).

According to the current 2018 ESC guidelines on the management of cardiovascular diseases during pregnancy, peripartum cardiomyopathy is defined as heart failure secondary to left ventricular dysfunction that develops toward the end of pregnancy or in the first few months postpartum. The left ventricle may not be dilated, but the left ventricular ejection fraction (LVEF) is usually <45%. An initial LVEF <30%, significant dilation (end-diastolic diameter ≥6 cm), and right ventricular involvement are associated with poor prognosis (5). Thorough history-taking is emphasized to exclude other causes of heart failure. Currently, diagnosis does not rely on strict time frames or echocardiographic criteria, reducing the risk of missing the diagnosis (1, 6).

The exact mechanism behind PPCM remains unclear. A multifactorial etiology is suggested, involving genetic and environmental factors (higher prevalence among African and African American populations, familial cases) (1, 7), infectious factors (viral genetic material has been detected in cardiomyocytes of 31% of patients with PPCM), and autoimmune processes (8). Particular attention has been given to a 16 kDa prolactin fragment generated during pregnancy, which exerts cardiotoxic effects. Its elevated levels in PPCM form the rationale for the use of prolactin inhibitors—most notably bromocriptine (class IIa recommendation, level C) (9, 10). Another contributing factor is an increase in soluble fms-like tyrosine kinase-1 (sFLT1), an antiangiogenic factor that, when overexpressed, leads to cardiomyocyte dysfunction. In such cases, administration of vascular endothelial growth factor (VEGF) has been considered, though efficacy has only been demonstrated when combined with bromocriptine (11, 12).

The clinical presentation of PPCM is nonspecific, commonly including dyspnea, edema, chest pain, fatigue, palpitations, and psychological symptoms (such as a sense of impending doom). In the presented case, the patient reported right upper quadrant pain with vomiting, initially suggesting cholecystitis. Symptoms typically appear within the first four months postpartum—as in this case—and less commonly late in pregnancy (1). The similarity of these symptoms to normal pregnancy-related complaints or other acute conditions (e.g., myocardial infarction, sepsis, amniotic fluid embolism, exacerbation of pre-existing heart failure) complicates diagnosis. The most frequently reported symptoms include dyspnea, fluid retention, excessive fatigue, and persistent cough (13).

Diagnosis of PPCM is generally made by excluding other causes (14). It should be considered in all peripartum women who experience a prolonged recovery to pre-pregnancy health status (15). Echocardiography remains the key diagnostic tool—a reduced LVEF <45% should prompt further investigation for PPCM (11). NT-proBNP levels, which are typically elevated in PPCM, should also be measured. Chest x-ray, ECG, cardiac MRI, and biopsy (if viral etiology is suspected) are complementary diagnostic tools (15, 16). Increasingly, additional biomarkers such as microRNA-146, endothelial cells, monocyte-derived microparticles, 16 kDa prolactin, and cathepsin D are being evaluated.

Therapeutic management of PPCM follows guidelines for acute heart failure and cardiogenic shock. Treatment should be continued for at least six months after normalization of left ventricular function, followed by gradual withdrawal, as abrupt discontinuation has been associated with relapses. Bromocriptine has been shown to improve left ventricular function, but its use requires concurrent anticoagulation with heparin. The BOARD acronym (Bromocriptine, Oral heart failure therapies, Anticoagulants, vasoRelaxing agents, Diuretics) summarizes the comprehensive approach to managing acute PPCM (6).

In the present case, the BOARD protocol was not fully implemented due to the patient's critical clinical status and the need for immediate life-saving intervention. Advanced cardiogenic shock and profound hemodynamic instability constituted contraindications to the use of bromocriptine. Oral heart failure therapies, including diuretics, were insufficient in the context of acute decompensation. Although anticoagulation was indicated (due to intracardiac thrombi and ECMO support), its administration was limited by severe coagulopathy and active bleeding at cannulation sites. Prior to transplantation, levosimendan was introduced for its positive inotropic and vasodilatory properties; however, classical vasodilators were not administered due to circulatory instability. Recurrent cardiac arrest despite maximal conservative therapy ultimately necessitated urgent heart transplantation.

In the presented case a combination of two mechanical circulatory support methods was employed: VA ECMO (veno-arterial extracorporeal membrane oxygenation) and IABP (intra-aortic balloon pump). No clinical improvement was achieved, and the presence of pedunculated thrombi in both ventricles precluded long-term circulatory support. The only life-saving option remaining was heart transplantation. A study by Cindy Song et al. analyzed the use of mechanical circulatory support as a bridge to transplantation in patients with PPCM. The findings support the safety and efficacy of this strategy, with early implementation of temporary mechanical circulatory support (tMCS) improving clinical outcomes (17).

Women with PPCM have a higher risk of transplant rejection and post-transplant mortality. Due to the high risk of recurrence, subsequent pregnancies are discouraged—at least for 12 months after transplantation (5, 14).

This case highlights that PPCM can progress rapidly, even in young women, and that prompt diagnosis and appropriate treatment are crucial for patient survival.

## Conclusions

PPCM is a rare yet potentially fatal form of heart failure. It is crucial to educate women about the risks associated with subsequent pregnancies, as there remains a risk of recurrent PPCM, which may worsen heart failure or lead to death.

In the late stages of PPCM, characterized by irreversible myocardial dysfunction, surgical intervention is the only viable treatment option. Orthotopic heart transplantation remains the definitive life-saving procedure for patients with the poorest prognosis.

## Data Availability

The original contributions presented in the study are included in the article/Supplementary Material, further inquiries can be directed to the corresponding authors.
